# Evaluation of V3–V4 and FL-16S rRNA amplicon sequencing approach for microbiota community analysis of tracheostomy aspirates

**DOI:** 10.1128/msphere.00388-25

**Published:** 2025-07-22

**Authors:** A. Gupta, V. S. Cooper, A. C. Zemke

**Affiliations:** 1 Division of Pulmonary, Allergy, Sleep and Critical Care Medicine, University of Pittsburgh School of Medicine 12317 , Pittsburgh, Pennsylvania, USA; 2 Microbiology and Molecular Genetics, University of Pittsburgh School of Medicine 12317 , Pittsburgh, Pennsylvania, USA; 3 Center for Evolutionary Biology and Medicine, University of Pittsburgh 6614, Pittsburgh, Pennsylvania, USA; University of Michigan-Ann Arbor, Ann Arbor, Michigan, USA

**Keywords:** full-length 16S, microbiome, amplicon sequencing, tracheostomy

## Abstract

Respiratory infections pose a significant risk for people requiring prolonged mechanical ventilation, yet limited information exists regarding the complex microbiome dynamics of people with tracheostomies during chronic critical illness. Oxford Nanopore Technologies (ONT) long-read sequencing allows for full-length 16S rRNA amplicon sequencing, providing enhanced species-level understanding of the respiratory microbiome. We validated ONT-based FL-16S amplicon sequencing for microbial insights from tracheal aspirates by comparing results with those of Illumina V3–V4 amplicon sequencing. Comparisons were made on a standardized microbial community and tracheal aspirates using multiple DNA extraction kits. Conventional short-read bioinformatic pipelines are suboptimal for processing longer, error-prone ONT reads. The Emu bioinformatics pipeline, specifically designed for ONT FL-16S reads, enhances the accuracy but necessitates validation for tracheal aspirates. In this study, we compared the analysis of FL-16S reads using Emu to the standardized V3–V4 read analysis with QIIME2. Our findings demonstrate that at the same sequencing read depth, FL-16S sequencing analysis with Emu yields comparable alpha and taxonomic diversity metrics, while providing superior species-level resolution compared to V3–V4 amplicon sequencing of tracheal aspirates. Our results show that tracheal aspirates during chronic critical illness are low-diversity samples, with most pathogenic genera represented by a single species. However, members of the oral microbiota *Streptococcus* and *Prevotella* are represented by multiple species.

**IMPORTANCE:**

The role of the respiratory microbiome in shaping outcomes for patients with chronic critical illness undergoing prolonged mechanical ventilation via a tracheostomy remains poorly understood, despite its potential to drive infections and complicate recovery. Current methods, such as short-read 16S rRNA sequencing, lack taxonomic resolution to track pathogens at the species level, limiting clinical insights. Our study addresses this gap by validating ONT-based full-length (FL)-16S rRNA sequencing, a method that achieves species-level taxonomic precision critical for analyzing complex respiratory microbiomes. We benchmarked the microbiome composition of tracheal aspirates from ONT FL-16S rRNA workflows against Illumina V3–V4 data to demonstrate that long-read sequencing delivers comparable diversity profiles while resolving species-level diversity of clinically relevant species and microbes associated with the oral microbiome.

## INTRODUCTION

The respiratory microbiome and its effect on respiratory health have been extensively characterized in both health and disease states. A healthy lower airway microbiota is constituted of traditionally oropharyngeal taxa such as *Streptococcus*, *Prevotella,* and *Veillonella*, albeit at low abundance (1, 2). Multiple studies have identified associations between the decrease in microbial alpha diversity and dominance of specific pathogens such as *Haemophilus* and *Pseudomonas* with disease severity in diverse illnesses including asthma, chronic obstructive pulmonary disease (COPD), and cystic fibrosis (CF) (3, 4). The pulmonary microbiome in critical illness has primarily been studied at the genus level to date in the contexts of adult respiratory distress syndrome (ARDS) or severe pneumonia. After the initiation of mechanical ventilation, the pulmonary microbiome rapidly shifts (5). Patients with ARDS and severe pneumonia are distinguishable from patients requiring mechanical ventilation for other reasons based on their bacterial community compositions (6, 7). Additionally, in critically ill patients requiring mechanical ventilation, low alpha diversity and higher bacterial burden can predict death or requirement of prolonged ventilation (6, 7). Studies on the microbiome in critical illness have largely focused on the first 1–2 weeks of mechanical ventilation. If respiratory failure persists, a tracheostomy is often placed to aid in weaning from mechanical ventilation. Clinically, this period is marked by episodes of pneumonia or tracheobronchitis, with a prevalence of 30%–40% (8, 9). However, little is known about the microbiome dynamics during recovery from prolonged mechanical ventilation or chronic critical illness.

The gold standard for microbiome characterization is to sequence one or more hypervariable regions within the conserved 16S ribosomal RNA gene. The 16S rRNA gene consists of nine constant and nine hypervariable regions (V1–V9) (10). Sequencing of one or more hypervariable regions is used to discriminate between different bacterial or archaeal taxa. The second-generation sequencing platforms such as Illumina that use short-read sequencing and generate ~150–300 bp long reads have been widely employed in microbiome studies for their high-throughput capabilities and low cost (11). The accuracy of this technology and the well-established and validated bioinformatic workflows, such as QIIME (12) and mothur (13), have certified its wide adoption in microbiome studies. However, amplification of a single or double hypervariable 16S rRNA region(s) with ~500 bp amplicon size lacks the taxonomic resolution required for species- or strain-level identification. In addition, estimates of taxonomic diversity can vary in the sample depending on the hypervariable region amplified (14, 15). Enhanced taxonomic resolution becomes crucial for clinical and epidemiological studies in which the exact identity of the infectious agent is required for dictating appropriate personal or public health measures.

An alternative approach to amplicon sequencing is shotgun sequencing, which provides a holistic view of all the genes present in the sample. Shotgun sequencing can be effective in providing species- or strain-level identity of a broad range of bacteria, fungi, and viruses (16, 17). Shotgun sequencing can also provide information about other functional genes in the community without introducing bias from PCR amplification. Although shotgun sequencing can provide a comprehensive understanding of microbial species and functional genes in a sample, the high sequencing costs can be a major deterrent in the study of respiratory microbiomes. These costs are further compounded by the presence of >99% host DNA in respiratory samples (18), which results in undersampling of microbial reads even at high sequencing depths. Experimental approaches targeting host DNA depletion can improve the proportion of microbial reads using shotgun sequencing; however, host depletion approaches can introduce a taxonomic bias to the results (18–20). A new generation of sequencing technologies is capable of generating longer reads ~10–100 kb. These long reads have thus allowed for the amplification of the entire 16S rRNA gene (FL-16S rRNA), providing enhanced information for species identification. The MinION sequencing platform provided by Oxford Nanopore Technologies (ONT) is portable, has a low capital cost, and can provide real-time data analysis (21). The long-read technologies have a higher sequencing error rate (4%–8%) (22) compared to the established Illumina sequencing (0.1%), and hence the proportion of microbiome studies employing ONT remains low compared to those employing Illumina sequencing (23). Recent improvements in the Nanopore sequencing chemistry (Q20+) and R10.4.1 flow cells can generate a read accuracy of ~99% (24, 25). Furthermore, specialized bioinformatics pipelines that can handle sequencing data with more errors, such as NanoClust (26), Emu (27), and BugSeq 16S (28), have also been developed to further increase the accuracy of ONT data for microbiome studies. Even though multiple studies have compared the use of ONT FL-16S and multiple bioinformatic pipelines for microbiome analysis of different human and environmental sample types (29–37), there are a few papers using ONT sequencing approaches in studies of lower respiratory samples (38, 39). Respiratory samples such as tracheal aspirates have very low microbial biomass, high abundance of host cells with their attendant nucleic acids, and can have very high viscosity, complicating sample handling (18, 19). The low microbial biomass makes it difficult to comprehensively capture the likely polymicrobial diversity of the sample, and hence the choice of DNA extraction method can change the observed microbial composition along with the choice of the sequencing technology.

The primary objective of this study is to validate the deployment of ONT FL-16S amplicon sequencing for understanding the microbial dynamics of tracheal aspirates collected from patients with chronic critical illness requiring tracheostomy.

## RESULTS

### Effect of the DNA extraction kit and sequencing method on the inferred composition of a reference standard bacterial community

**Fig 1:**
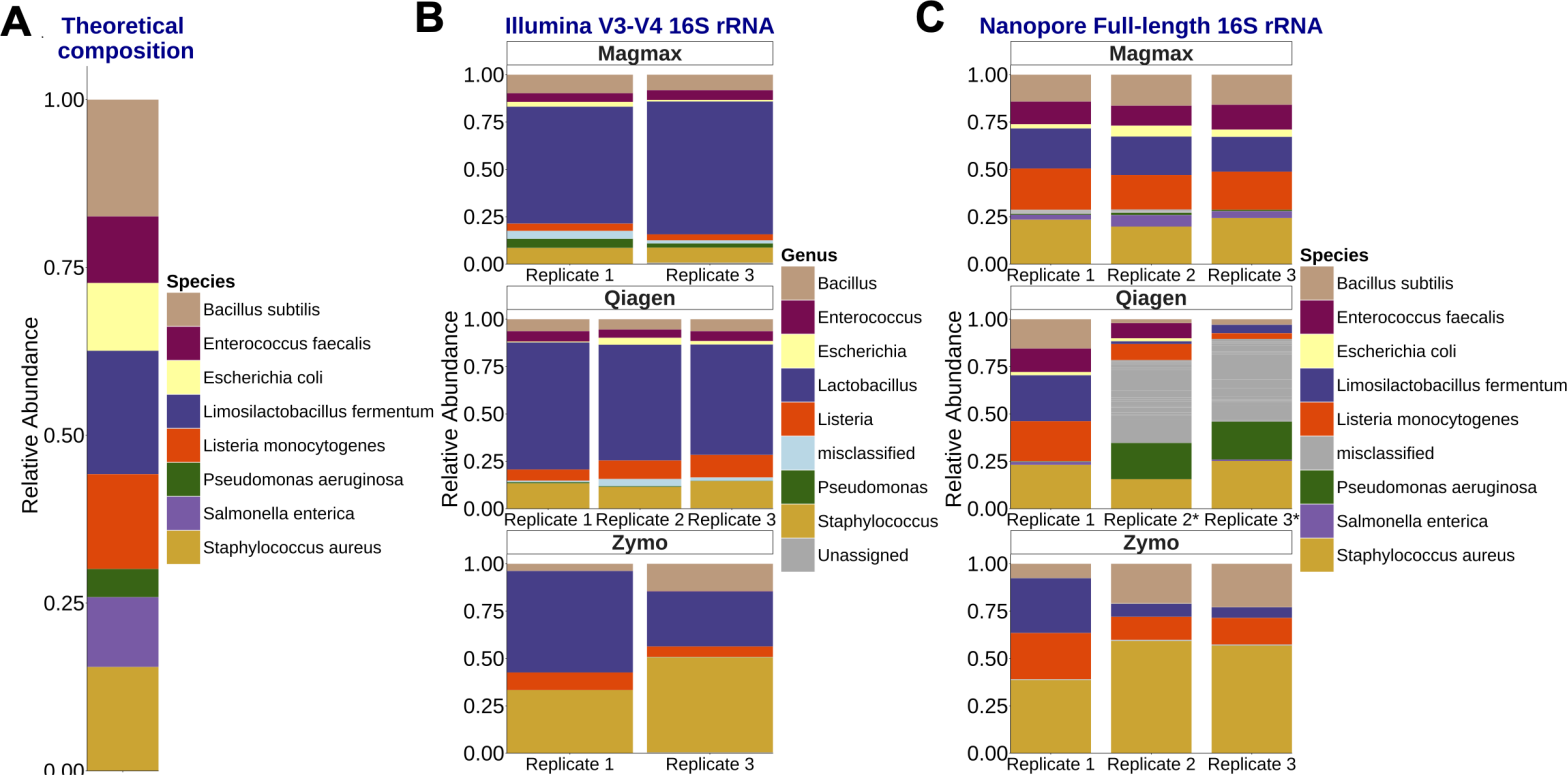
Full-length 16S sequencing effectively captures all the species in the mock community. (**A**) The expected composition of the eight bacterial species present in the mock community. DNA was extracted from three aliquots of the Zymobiomics DNA standard using each of the selected kits. The same extracted DNA was amplified and sequenced for either the (**B**) V3–V4 hypervariable region using Illumina sequencing or the (**C**) full-length 16S rRNA using Nanopore sequencing, followed by taxonomic profiling using QIIME2 or Emu, respectively. Samples extracted using the host-depletion kit (Zymo) depleted certain gram-negative species from the community. *indicates that the sample had low sequencing output, i.e., less than 5,000 reads. Replicate 2 from the MagMax and Zymo DNA extraction kits had less than 500 sequencing reads from Illumina sequencing and was excluded from the analysis.

We first evaluated the taxonomic bias of each of the DNA extraction and sequencing methods using a reference community of known bacterial composition (Fig. 1A). We extracted DNA of an aqueous solution of the reference community to determine which method combination had the least bias, gave reliable results, and allowed the greatest species resolution. QIAamp BiOstic and MagMax kits yielded similar DNA concentrations (median 12 µg/mL). DNA yield from the Zymo HostZero kit using the nuclease depletion steps as described in the manufacturer’s protocol yielded DNA concentrations below 0.1 ng/µL, which was inadequate for subsequent analysis.

Each of the three test kits is effective in lysing gram-positive bacteria, as observed by V3–V4 amplicon sequencing. Approximately 90% of bacteria were correctly classified at the genus level, with the exception of *Salmonella*. However, the relative abundance of the sequenced community deviated from the theoretical composition (Fig. 1B). Amplification with 27 F-1492R primers and FL-16S rRNA Nanopore sequencing captured microbial diversity comparable to the V3–V4 approach but achieved species-level resolution (Fig. 1C). The mock community extracted with the MagMax kit closely resembled the theoretical composition, capturing all species with high accuracy (intraclass correlation coefficient ~0.85, Table S1). The Zymo kit host depletion protocol led to the loss of certain gram-negative bacteria, regardless of the sequencing method. Furthermore, we compared the taxonomic assignment of short- and long-read sequences of the mock community using each of the bioinformatic pipelines and the different databases (Fig. S1). The Emu pipeline uses an approximation-based read mapping algorithm, which is incompatible for the analysis of highly similar regions, such as short V3–V4 amplicons (27). Hence, even though Emu can map most of the V3–V4 short reads to a database, the taxonomic assignment is unclassified (Fig. 1C). Emu also uses a curated database containing entries from the NCBI RefSeq and rrnDB without duplicate entries. Unlike the database SILVA, which is not curated for species-level information (40) and results in higher false positives, the prebuilt Emu database provides greater taxonomic rigor.

### Comparison of V3–V4 Illumina and FL-16S rRNA Nanopore microbial diversity classification in tracheal aspirates

We next tested the efficacy of DNA extraction and amplicon sequencing of five tracheal aspirates collected from our study participants. One of the samples had less than 5 k reads for each DNA extraction and was dropped from the analysis. We first estimated the ratio of microbial to host DNA of each sample using quantitative-PCR (qPCR) of 16S and 18S gene copies. DNA from MagMax and Qiagen kits contained less than 1% microbial DNA (Fig. S2). Despite DNA concentrations below 0.1 ng/µL, DNA extracted using the Zymo kit contained 50%–90% microbial DNA (Fig. S2).

**Fig 2:**
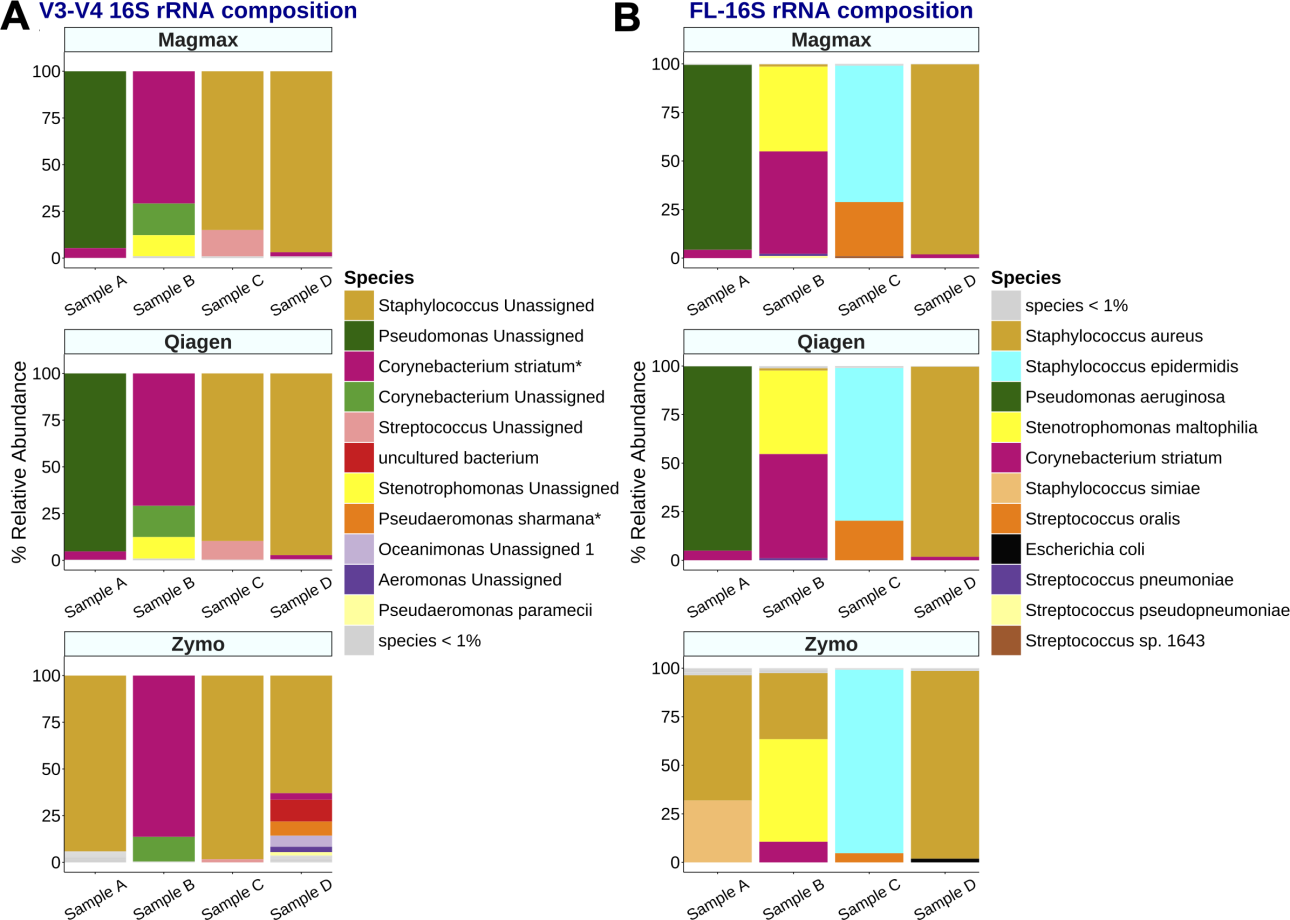
FL-16S rRNA sequencing approach improves the resolution of bacterial species in tracheal aspirates. Participant tracheal aspirate samples were extracted using each of the selected DNA extraction kits, followed by sequencing of the (**A**) V3–V4 hypervariable region or (**B**) full-length 16S rRNA and analysis using QIIME2 or Emu, respectively. FL-16S rRNA provides species-level taxonomic detail for microbes present in the clinical samples. All samples underwent DNA extraction in triplicates. Data representative of a single replicate. * indicates <90% confidence in species-level classification.

For each aspirate, community composition was similar between samples extracted with the Qiagen and MagMax kits (Fig. 2). In contrast, samples extracted with the Zymo kit showed substantial variation, especially when dominated by gram-negative organisms like *Pseudomonas aeruginosa*. The FL-16S sequencing profile from tracheal secretions closely matched the microbial composition observed with V3–V4 sequencing. The MagMax method reliably yielded quantifiable DNA, sufficient FL-16S and V3–V4 reads, and comparable microbial data to other extraction kits. Therefore, we chose the MagMax kit for subsequent comparative studies of the microbiota from tracheal aspirates using both sequencing platforms.

**Fig 3:**
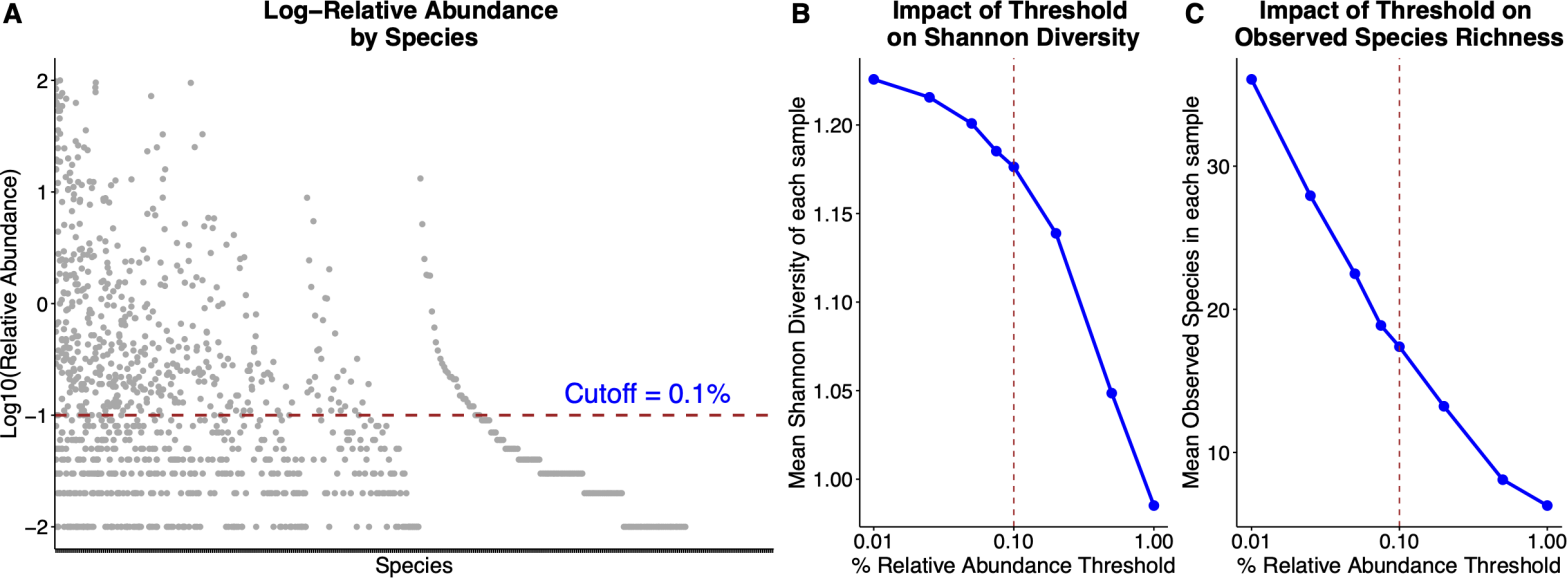
Filtering of low-abundance species from ONT sequencing analysis. The probabilistic approach of the Emu pipeline generates a long tail of low abundance species. (**A**) To minimize the effects of rare species on community comparisons, we chose a relative abundance cutoff of 0.1% to evaluate the composition and diversity. The median count of species with a relative abundance less than 0.1% for a given sample was 18, whereas the number of species above 0.1% relative abundance varied from 1 to 65. At the relative abundance threshold of 0.1% (**B**), the mean Shannon diversity of each tracheal aspirate is 1.17, and (**C**) the mean number of observed species in each sample is 17.

We sequenced the DNA extracted from 27 additional tracheal aspirates (for a total of 31) to compare the microbiome profiles generated using V3–V4 sequencing and FL-16S rRNA sequencing. The extraction, amplification, and sequencing processes were reliable, with most samples yielding adequate data for analysis (Table S2). The genus-level microbiome composition of FL-16S rRNA sequenced samples is similar to the short-read V3–V4 sequencing (Fig. S3), underscoring the analytical validity of the FL-16S rRNA microbiome profiling technique for use with tracheal aspirates. Since the bioinformatics pipeline for Nanopore reads, Emu, utilizes an expectation-maximization algorithm to correct for sequencing errors, the taxonomic assignment results in a long tail of low-abundance species. While not needed for the analysis of a mock community, these low-abundance, low-confidence assignments need to be handled consistently in samples with higher biologic complexity. We used a relative abundance of 0.1% as a cutoff to filter the low abundance species after observing the overall abundance distribution of all the species in the 31 samples (Fig. 3; Fig. S5).

**Fig 4:**
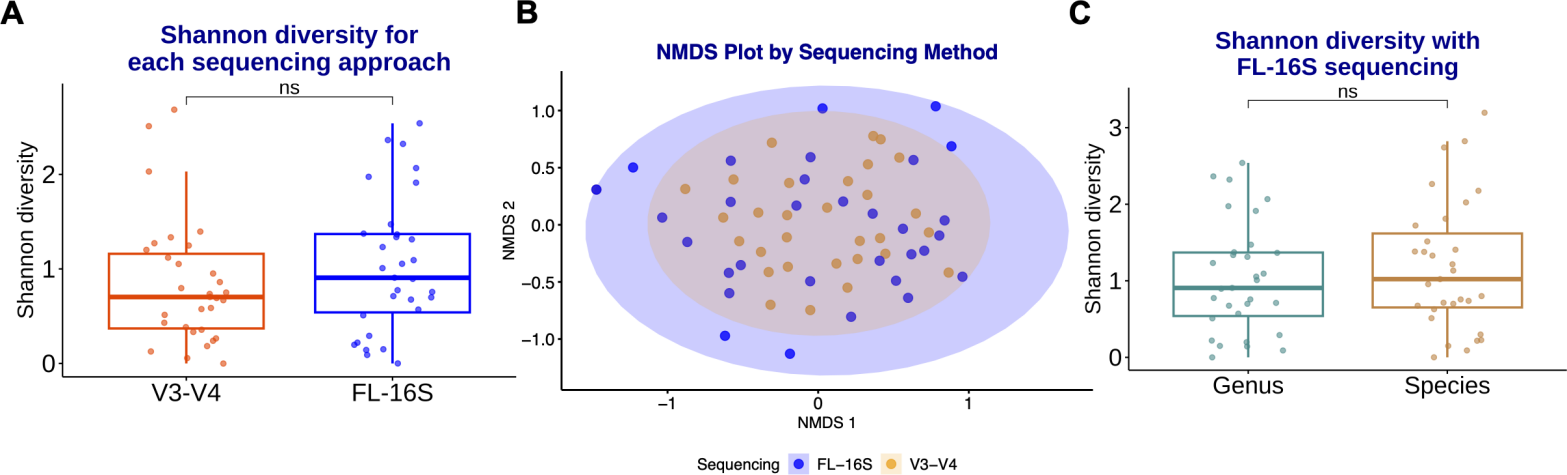
Comparison of community diversity inferred by FL-16S rRNA sequencing or V3–V4 sequencing for 31 tracheal aspirate samples. (**A**) Shannon diversity metrics comparing sequencing approaches across 31 samples. No statistically significant differences were observed between approaches. (**B**) Non-metric multidimensional scaling (NMDS) plot of the Bray-Curtis dissimilarity index between V3–V4 and FL-16S rRNA sequencing. FL-16S sequencing results were compositionally similar to V3–V4 samples (PERMANOVA, *P*-value = 0.84). (**C**) Shannon diversity measure at the genus vs species taxonomic level with the ONT FL-16S rRNA sequencing approach. No statistically significant increase in species-level Shannon diversity was observed, suggesting most genera in our samples are represented by a single species.

Both amplicon approaches yielded similar alpha and beta diversities at the genus level (Fig. 4A and B). Statistical analysis of reads not rarefied to 10,000 also gave similar alpha and beta diversity measurements (Fig. S4).

Interestingly, despite the increased taxonomic resolution with FL-16S rRNA, we do not observe a statistically significant increase in the Shannon diversity of species compared to the Shannon diversity of the genera present in the data set (Fig. 4C).

**Fig 5:**
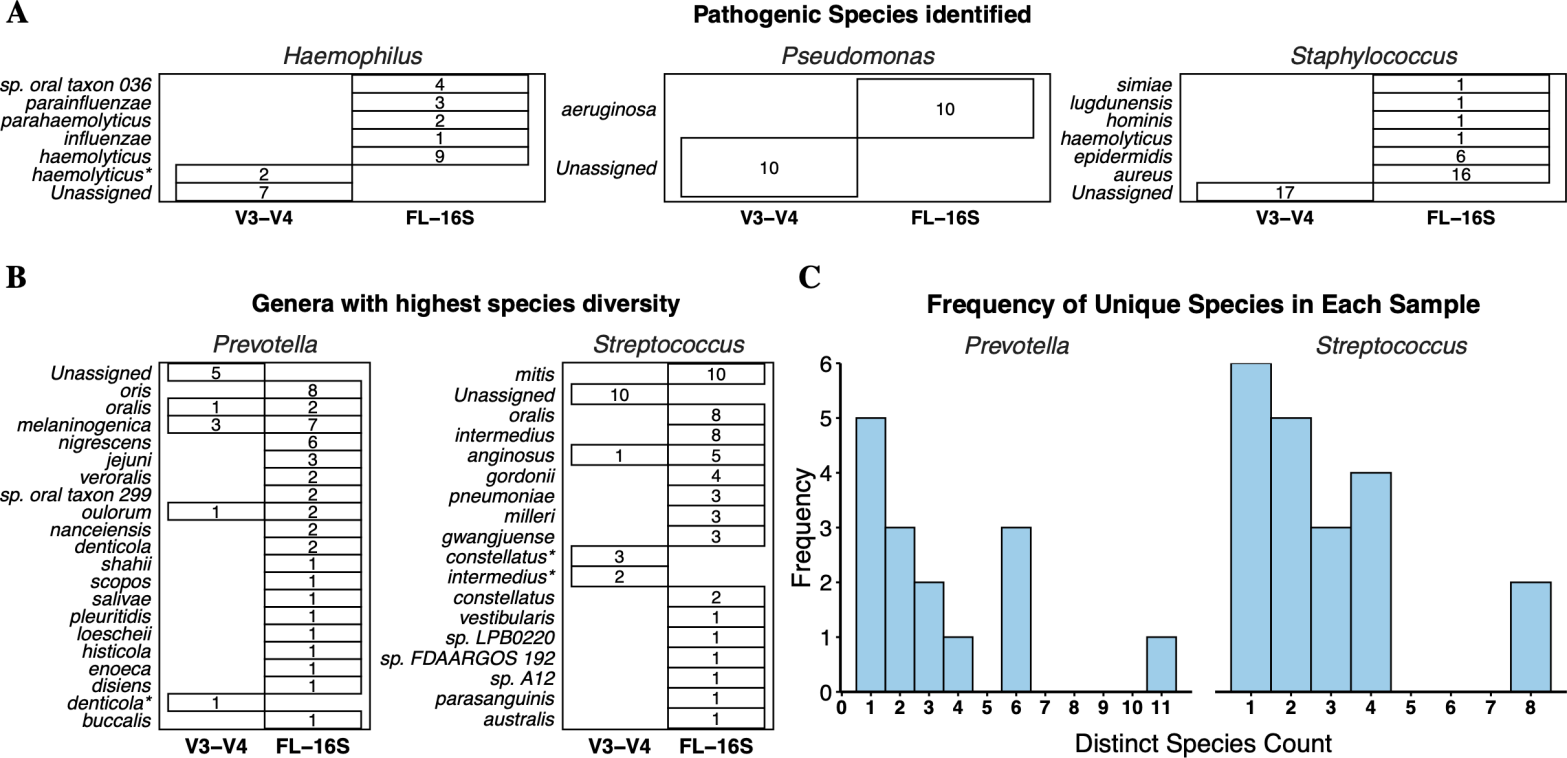
FL-16S rRNA identifies the presence of multiple commensal species per sample. Numbers indicate sample counts (of 31 samples) in which that species was identified at a relative abundance of >0.1%. (**A**) Species identification of common respiratory pathogens in the tracheal aspirates shows dominance by a single species from the specified genera. (**B**) *Prevotella* and *Streptococcus* show the maximum species diversity. (**C**) More than 50% of samples containing the genera *Prevotella* and *Streptococcus* contain multiple species of each genus.

Finally, we compared the taxonomic results from the sequencing techniques for distinctions in specific genera of clinical interest. Our data revealed a high abundance of *Staphylococcus* in several samples. Using the FL-16S rRNA sequencing approach, we were able to classify these abundant reads as belonging to the pathogenic species *Staphylococcus aureus* (*S. aureus*) in most samples. However, in some samples, this genus comprised multiple distinct less frequently pathogenic species such as *S. epidermidis* and *S. simiae*, demonstrating the potential of discriminating between commensal and pathogenic species using FL-16S rRNA sequencing (Fig. 5A). Using this method, we were also able to classify and identify other pathogenic species such as *Pseudomonas aeruginosa*, *Klebsiella oxytoca*, *Escherichia coli*, *Enterococcus faecalis, and Acinetobacter baumannii* in our samples (41) (Fig. 5A; Fig. S5). Although most genera are represented by a single species, certain genera such as *Streptococcus* and *Prevotella* are present as a collection of multiple different species in a given sample (Fig. 5B and C). These genera are commonly found in the oral microbiome and are often associated with a healthy lung microbiome (4, 42).

## DISCUSSION

Clinical samples are complex in their composition, available in limited quantities, and can be very difficult to obtain, thus requiring validated analytical techniques that reduce loss of relevant information from the samples. In this study, we tested multiple DNA extraction approaches that could be employed for tracheal aspirates and validated the use of FL-16S amplicon sequencing to retrieve valuable insights into the pathogens present and the broader microbial ecology within these samples.

We tested three different DNA extraction kits, Qiagen QIAmp, Zymo HostZero, and ThermoFisher Magmax Microbiome, designed to address the complex characteristics of tracheal secretions to identify the least biased and most consistent approach for DNA extraction from tracheal aspirates. Using a mock community, we assessed the extraction efficacy and sequencing results from V3–V4 and FL-16S amplicon sequencing. We next contrasted the microbiome composition of tracheal secretions determined by ONT FL-16S amplicon sequencing analyzed using the error handling pipeline Emu with results from the conventional V3–V4 amplicon sequencing analyzed using QIIME2. Benchmarking ONT FL-16S against V3–V4 amplicon sequencing showed that ONT FL-16S sequencing provides accurate microbiome profiles from tracheal aspirates.

The ONT platform has significant advantages for microbiome analysis due to its portability, real-time and rapid sequencing, and low capital costs (21). These features of ONT-based sequencing make it particularly appealing for infectious disease diagnostics. Researchers have presented preliminary evidence for the application of ONT-based 16S sequencing for the rapid detection of pathogens in less than 2 hours (43, 44). The R10.4.1 flow cells combined with updated chemistries and advanced basecalling algorithms have achieved substantial improvements in read accuracy, an initial implementation barrier (45). Studies have reported that raw read accuracies with R10.4.1 can reach approximately 99%, comparable to Illumina’s performance (25). Our findings also demonstrate that technical advances in both sequencing and analysis of FL-16S rRNA now yield reliability comparable to that of widely established Illumina sequencing methods, validating the use of ONT-based FL-16S rRNA amplicon sequencing for undiluted respiratory specimen analysis.

Another factor that limits the employment of FL-16S rRNA for microbiome studies was the lack of a single widely used and simplified data analysis pipeline. The EPI2ME software developed by Oxford Nanopore provides a simple, user-friendly interface, along with real-time analysis for 16S amplicon analysis (46). However, it provides limited features for filtering and error correction of ONT reads; thus, it delivers less accurate taxonomic assignments.

The recent 16S data analysis pipeline for ONT sequences, Emu, uses the minimap2 read aligner for mapping and an expectation-maximization algorithm to correct the error rate for optimized results (27). Although Emu can reduce the number of false positives in the sample set, the optimal abundance cutoff to distinguish noise from true assignments is randomly chosen by the developers of the pipeline. This feature can limit the detection of ultra-low abundance species in the sample. Although the Emu pipeline enforces a minimum threshold of 10 reads per 1,000 total reads, we applied an additional 0.1% relative abundance filter to minimize noise and highlight dominant clinically relevant taxa in these low-complexity samples (Fig. 3). This eliminates the long tail from the distribution of taxa abundance and reduces the effects of rare community members on sample comparisons.

We show that commercial microbial DNA extraction kits such as ThermoFisher-Magmax and Qiagen-BioAmp kits are effective in capturing 100% of the microbial diversity of the mock communities, and they performed well on human tracheal aspirates, despite the abundance of host DNA (47). In metagenomic sequencing, which does not include an amplification step, the abundance of host DNA requires massively increased sequencing depth to obtain the same bacterial genomic resolution. To overcome this barrier, selective DNA degradation approaches such as benzonase-based host DNA degradation have been employed to enrich microbial DNA (48). However, in our hands, a kit that included host degradation yielded very low total DNA, and more concerningly, showed high bias with selective loss of gram-negative species. Because FL-16S includes an amplification step, host depletion was not required for adequate technical performance. A recent study evaluated multiple host-DNA depletion methodologies for various respiratory samples and demonstrated that host depletion changed the microbiome composition of sputum samples, thus underscoring that the optimal DNA extraction approach will vary by sample type (19).

We also evaluated whether the choice of the PCR-amplified 16S rRNA region and corresponding sequencing technology affects sequencing yields and taxonomic accuracy. In our analyses of the mock community using V3–V4 Illumina sequencing, *Lactobacillus* was overrepresented. This variation could arise due to potential bias in the specificity of primers and/or stringency of PCR conditions. Conversely, PCR amplification of FL-16S rRNA reduced bias and also achieved higher taxonomic classification for both the mock community samples and tracheal aspirates.

Accurate taxonomic classification depends on high-quality input databases. To evaluate reference community sequencing data, we compared the accuracy of taxonomic assignments using two popular databases: SILVA and a curated database containing entries from NCBI RefSeq and rrnDB. The SILVA database is widely popular due to its large database size, regular release cycles, and curated taxonomy. However, SILVA entries are not curated at the species level (40, 49, 50). Even with FL-16S rRNA sequences, we observe ~20% of read misclassifications using the SILVA database. Whereas the NCBI RefSeq and rrnDB databases cover a less diverse sequence space than the SILVA database, these databases are highly curated and accurate for species-level taxonomy. The small database size of the NCBI RefSeq and rrnDB could be limiting for microbial characterizations of less-studied environments or unknown microbes. However, the microbial composition of our clinical samples is well-represented in the smaller database, and they provided better classification accuracy.

Our pilot study involved 31 tracheal aspirates collected from people requiring prolonged mechanical ventilation following an incident episode of critical illness. Analysis of these microbiomes revealed that tracheal secretions are low-alpha diversity samples, mostly dominated by one or two genera. Furthermore, most genera are represented by a single species, creating similar taxonomic profiles between V3–V4 and FL-16S sequencing. Illumina V3–V4 sequencing proved equally informative as FL-16S sequencing for pathogen identification in these specimens, as only one species each from the genus *Pseudomonas* and *Staphylococcus* was identified. The low alpha diversity seen in these samples and the presence of known respiratory pathogens is comparable to the limited airway microbial diversity observed in other lung diseases such as cystic fibrosis, COPD, and ventilator-associated pneumonia (2, 7, 51).

Nonetheless, the FL-16S approach might be preferred in several scenarios in which species-level resolution is critical. First, anatomic locations where multiple species in the same genera are common and have different biologic implications. The upper respiratory tract (nose and paranasal sinuses) can harbor distinct *Staphylococcus* species with divergent clinical outcomes (52, 53). Here, the enhanced taxonomic resolution achieved through FL-16S rRNA sequencing can facilitate improved understanding of airway ecology. Second, FL-16S rRNA approaches are valuable for cohorts with many healthy individuals or lung disease states with higher alpha diversity. A recent study demonstrated its utility in non-CF bronchiectasis by characterizing diverse microbiomes and differentiating pathogenic *Proteobacteria* like *Haemophilus influenzae* from other species from the same genus such as *H. parainfluenzae* and *H. haemolyticus* (39). By examining different disease states and respiratory samples, researchers also identified multiple species from the genus *Streptococcus*, *Prevotella*, and *Veillonella* that correlated with a healthy microbiome (38, 39). These findings in parallel work in CF where people with less severe disease more often possess more diverse microbiomes that are dominated by *Streptococcus* and *Veillonella* (4). Third, the FL-16S rRNA approach might be especially informative in evaluating the presence and role of oral microbiota when pathologic aspiration of oral contents is suspected, such as in individuals with tracheostomies. The oral microbiome comprises a large number of closely related species of *Streptococcus*. However, oral genera such as *Streptococci* are poorly differentiated using the V3–V4 hypervariable region, and thus, FL-16S rRNA can provide better species differentiation for oral taxa (42, 54). Indeed, a recent study demonstrated enhanced resolution of oral bacterial phylotypes by ONT and superior taxonomic accuracy using the Emu pipeline for saliva samples (29).

Although Emu provided accurate taxonomic classification of our samples, we did not compare FL-16S rRNA sequencing data with those of other Nanopore specific tools such as NanoClust, which has similar performance accuracy as Emu (27), and EPI2ME which provides a user-friendly interface for wider access.

In conclusion, the present investigation validates the use of FL-16S ONT sequencing along with the data analytical pipeline Emu for use with tracheal aspirates. Our study demonstrates that sequencing data obtained via V3–V4 Illumina and FL-16S ONT platforms exhibit high concordance in profiling the microbial communities of tracheal aspirates with low diversity. DNA extraction using the MagMax kit and amplification of the full-length 16S rRNA gene provide reproducible and taxonomic resolution useful for better understanding the bacterial ecology of the respiratory tract in health and disease.

## MATERIALS AND METHODS

### Sample collection

Samples were obtained from participants enrolled in an observational cohort study of people with a tracheostomy in place recovering after critical illness (IRB STUDY20110443) in Pennsylvania, PA. Patients or their legally authorized representatives consented to participation in the study. Tracheal aspirates were collected using a new sterile suction catheter without the addition of saline. Samples were aliquoted and frozen at −80°C until DNA extraction in sterile vials. The sample set consists of 32 tracheal aspirates collected from 12 participants, with most participants contributing at least two samples. Samples from any single participant were collected a minimum of 1 week apart.

### Choice of DNA extraction methods for tracheal secretions

Our samples, i.e., respiratory tracheal aspirates, have four main characteristics: they have low volume, are viscous, they have a high concentration of human neutrophils, and they tend to have low microbial biomass. To work with these characteristics of the tracheal aspirates, we resuspended the sample in Sputasol to lower the viscosity of the sample. We chose three DNA extraction kits with different features to evaluate which DNA extraction method works best for these samples. As our samples have a high concentration of host DNA, we chose the Zymo HostZero (D4310) kit to evaluate the impact of reducing host DNA for microbial identification. Depleting host DNA is critical for metagenomic sequencing as high levels of host DNA can overwhelm the output sequencing reads, resulting in insufficient microbial reads for valuable metagenomic data. As the tracheal aspirates are of low microbial biomass, we tested the Qiagen Bacteriome kit (12240-50) kit to evaluate its efficiency in increasing microbial DNA yield. Tracheal aspirates isolated from tracheostomy tubes can be polymicrobial in nature. Hence, the ThermoFisher Magmax microbiome kit (A42358) kit was examined to evaluate its efficiency in isolating DNA from diverse microbial communities.

### Microbial community standard

The ZymoBIOMICS microbial standard (D6300) contains microbial community comprising hard-to-lyse gram-positive microbes and gram-negative microbes and includes two common respiratory pathogens, *Pseudomonas aeruginosa* and *Staphylococcus aureus,* along with a couple of tough-to-lyse yeasts. A volume of 200 µL of this community standard was extracted using each of the three DNA extraction kits in triplicates to evaluate the DNA extraction efficiency of each of these kits.

### DNA extraction from tracheal secretions

Five tracheal aspirates were swabbed followed by resuspension of the swab in 7.5% sputasol solution. Each sputasol resuspended solution was aliquoted into nine tubes for DNA extraction by three different extraction methods in triplicate. All tubes were incubated at room temperature for 1 hour to facilitate hypotonic lysis of mammalian cells. Cells were then centrifuged at 13,000 rpm for 10 minutes to pellet the bacterial cells, followed by the addition of lysis buffer included in the respective kits. For DNA extraction using the MagMax kit, all 27 additional tracheal aspirate swab samples were incubated with distilled water and 7.5% sputasol for 1 hour at room temperature to facilitate hypotonic lysis of mammalian cells, followed by the manufacturer’s instructions for DNA extraction. Extracted DNA from each kit was eluted in nuclease-free water. The DNA concentration was quantified using a high-sensitivity dsDNA assay kit on a Qubit fluorometer.

### Quantitative real-time PCR to evaluate the 18S/16S rRNA ratio and percentage of bacterial DNA

To estimate the ratio of bacterial DNA to host DNA in the tracheal sections, quantitative real-time PCR was performed using the extracted DNA. The 16S rRNA and 18S rRNA genes were used as target genes for amplification. PCR was performed using the bacterial universal primers for 16S rRNA (5′CCTACGGGAGGCAGCAG-3′ and 5′-ATTACCGCGGCTGCTGG-3′) and human 18S rRNA primers (5′-GGTGGTGCCCTTCCGTCA-3′ and 5′-CGA TGCGGCGGCGTTATT-3′), respectively (55). A standard curve was generated using a known concentration of *Pseudomonas aeruginosa* genomic DNA and human carcinoma cell DNA, amplified with the same 16S and 18S rRNA primers to calculate the copy numbers of 16S rRNA and 18S rRNA, respectively. The ratio of 16S/18S copy number was calculated as described in Heravi et al. (55).

### DNA sequencing and analysis

For FL-16S amplicon sequencing, the extracted DNA was sent to a sequence provider, SeqCoast Genomics, where the samples were PCR-amplified using the primers 27F 5′ AGRGTTYGATYMTGGCTCAG 3′ and 1492R 5′ RGYTACCTTGTTACGACTT 3′. PCRs were purified using an SPRI bead cleanup and then used as input for the Oxford Nanopore Technologies (ONT) SQK-NBD114 native barcoding kit. Sequencing was performed on the GridION platform using a FLOW-MIN114 Spot-ON Flow Cell, R10 version with a translocation speed of 400 bps. Base calling was performed on the GridION using the super-accurate base calling model, Guppy, 7.0.9. The ONT sequencing data were filtered using Seqkit (v. 0.12.0) to filter sequencing reads of minimum quality 10 and length in the range of 1,200–1,800 base pairs. Chimera reads were removed using the vsearch-2.6.0 package. The filtered reads were then classified using the Emu pipeline (27) with Emu’s custom database comprising rrnDB version 5.6 (56) and NCBI 16S RefSeq (57). The classified data were then imported into R, where the data were rarefied using the phyloseq package.

For 16S V3–V4 hypervariable region sequencing using Illumina technology, the samples were prepared using the Zymo Quick-16S Plus NGS Library Prep Kit with unique dual indexes (D6421-PS1). Sequencing was performed on the Illumina MiSeq platform using a MiSeq reagent kit v3 to produce 300 bp single-end reads.

Hypervariable amplicon sequencing data were imported into QIIME2 v2022.11 (58) and analyzed using the “Demultiplexed Single-End” option. The reads were denoised, and the read ends were trimmed from 5 to 280 base pairs to remove low-quality base pairs using the DADA2 plug-in (59). Chimeric sequences were removed using the VSEARCH uchime-denovo plug-in (60). Samples were rarefied to an optimal depth of 10,000 to maintain the maximum number of observed features before classifying taxa using the SILVA 138 99% OTUs full-length sequence rRNA database (50).

Feature data generated by QIIME2 were then imported into R for further analysis. Non-bacterial taxa were filtered from the data set, and duplicate genera were agglomerated using the function “tax_glom” and specifying genus as the taxonomic rank. Taxa labeled “unassigned” at the genus level were extracted, and their V3–V4 variable region amplicons were extracted from the representative sequence file. Unassigned 16S sequences were queried using BLAST (61), and sequences with a >95% similarity to a given organism were manually assigned to that genus. Relative abundance per ASV per sample was calculated, and these values were used for further analysis.

### Statistical analysis

The samples from both V3–V4 and FL-16S rRNA sequencing were rarefied to 10,000 reads. The “phyloseq” package (62) in R was used to calculate the alpha and beta diversities of samples for both Illumina and Nanopore sequencing data. The Bray-Curtis and unweighted unifrac distance NMDS analyses were carried out using the phyloseq package in R with default settings. The ggplot2 package (63) in R was used to plot taxonomic abundances and NMDS measures. The “irr” package (64) was used to calculate the intra-class correlation coefficients (ICC) between the sequenced mock community and theoretical composition for each of the DNA extraction kits.

## Data Availability

Raw sequencing data can be found at NCBI BioProject PRJNA1279269.

## SUPPLEMENTAL MATERIAL

Supplemental material msphere.00388-25-s0001.pdf Supplemental table and figures.

ASM does not own the copyrights to Supplemental Material that may be linked to, or accessed through, an article. The authors have granted ASM a non-exclusive, world-wide license to publish the Supplemental Material files. Please contact the corresponding author directly for reuse.
