# Supplementary material for: Evaluation of V3–V4 and FL-16S rRNA amplicon sequencing approach for microbiota community analysis of tracheostomy aspirates
Source: mSphere. 2025 Jul 22;10(8):e00388-25. doi: 10.1128/msphere.00388-25 (PMC12379586; doi:10.1128/msphere.00388-25)
Supplement: Supplemental material — Supplemental table and figures. [file msphere.00388-25-s0001.pdf]

Supporting Information for

Evaluation of V3-V4 and FL-16S rRNA amplicon sequencing approach for microbiota community analysis of tracheostomy aspirates

Anshika Gupta, Vaughn S. Cooper, Anna C. Zemke

Corresponding author: Anna C. Zemke
Email: zemkea@upmc.edu

This PDF file includes:

Table S1 to S2
Figures S1 to S5

Table of contents:

p.3. **Figure S1:** Taxonomic classification of FL-16S and V3-V4 rRNA sequencing data of mock community DNA using different databases

p.4. **Table S1:** Intraclass correlation for DNA extraction using each of the test kits.

p.5. **Figure S2:** Quantification of microbe:host DNA

p.6. **Table S2:** Distribution of read counts before and after quality filtering.

p.7. **Figure S3:** Genus-level taxonomic classification of tracheal aspirates.

p.8. **Figure S4:** Microbiome diversity analysis without rarefaction

p.9. **Figure S5:** All species detected in tracheal aspirates using FL-16S rRNA sequencing

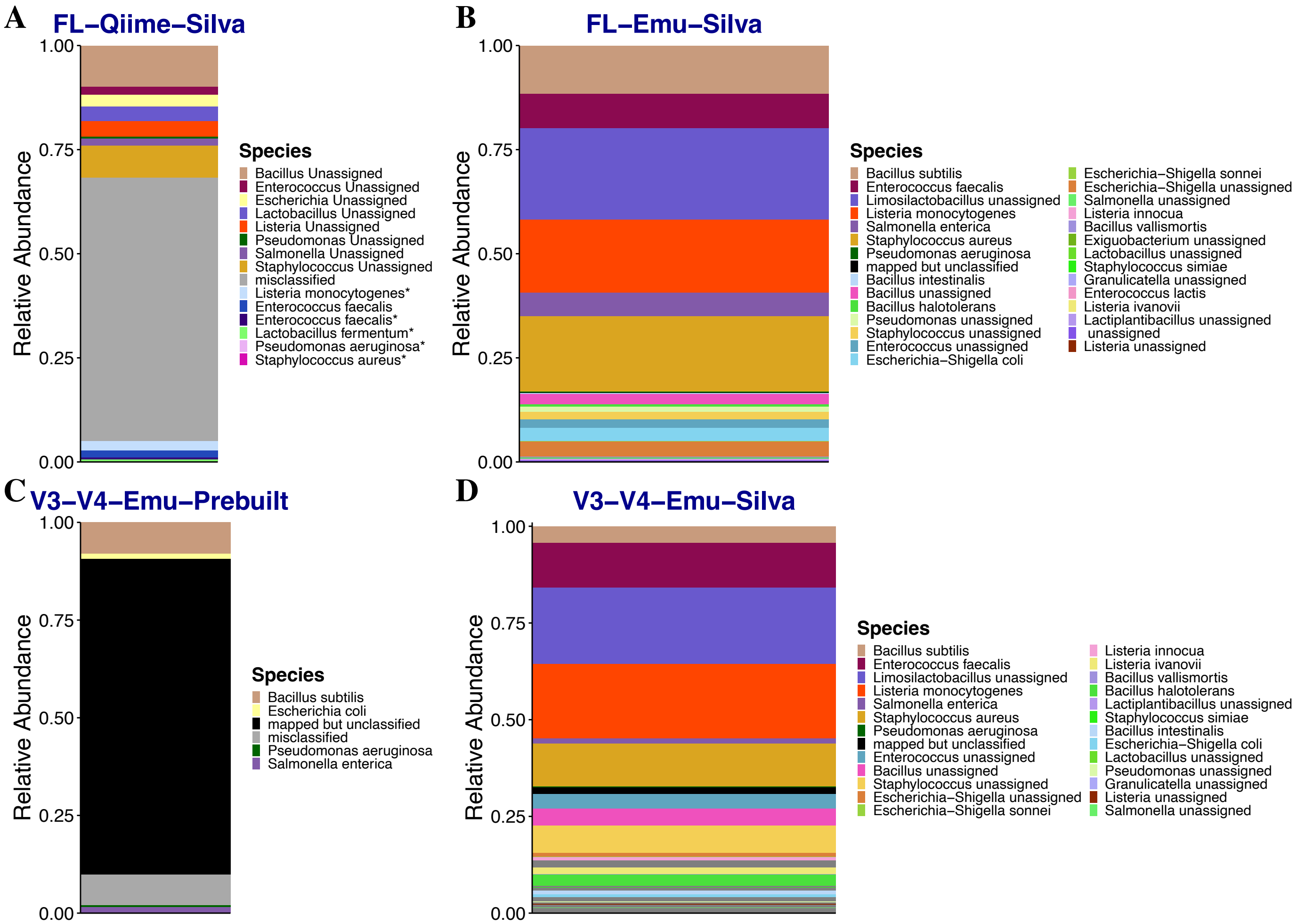

Figure S1: Taxonomic classification of FL-16S and V3-V4 rRNA sequencing data of mock community DNA using different databases. Classification of (A) FL-16S rRNA using the QIIME2 pipeline and using a classifier trained on the SILVA database (B) FL-16S rRNA using the Emu pipeline and mapping to the SILVA database (C) V3-V4 16S rRNA using the Emu pipeline and mapped to the Emu prebuilt database (NCBI and rrnDB) (D) V3-V4 16S rRNA using the Emu pipeline and mapped to the SILVA database. All that did not belong to the original mock community composition were clubbed under 'misclassified' in panels A and C. The Emu pipeline employs stringent parameters for read classification. The 'mapped but

unclassified' represent reads that had more than one hits to the database and could not be accurately assigned to a species present in the database.

Table S1: Intraclass correlation for DNA extraction of the mock community using each of the test kits

**Kit**	**Replicate**	**Intraclass correlation (ICC)**	**p_value**
Magmax	Replicate 1	0.89	7.67e-11
Magmax	Replicate 2	0.96	2.13e-14
Magmax	Replicate 3	0.70	0.023
Qiagen	Replicate 1	0.79	0.003
Qiagen	Replicate 2	0.32	0.051
Qiagen	Replicate 3	0.33	0.067
Zymo	Replicate 1	0.69	0.0005
Zymo	Replicate 2	0.48	0.040
Zymo	Replicate 3	0.50	0.011

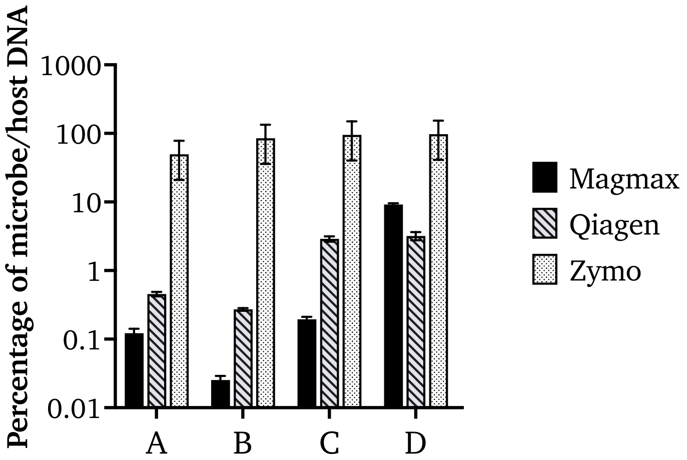

Figure S2: Quantification of microbe:host DNA. The HostZero depletion kits reduces the host DNA in DNA extraction from tracheal aspirates. qPCR analysis was used to estimate ratio of bacterial/host DNA in extracted DNA from four tracheal aspirates with each of the extraction kits in triplicates. The extracted DNA was amplified using 16S and 18S rRNA gene primers to estimate the number of 16S and 18S gene copies in the sample. The microbiome composition of these samples is presented in Figure 2 of the main text.

Table S2: Distribution of read counts before and after quality filtering.

	**Minimum number of reads**	**Median number of reads**	**Maximum number of reads**
ONT pre-QC	18863	164449	497866
ONT post-QC	1560	112239	294112
Illumina (pre-and post-QC)	3374	163433	274699

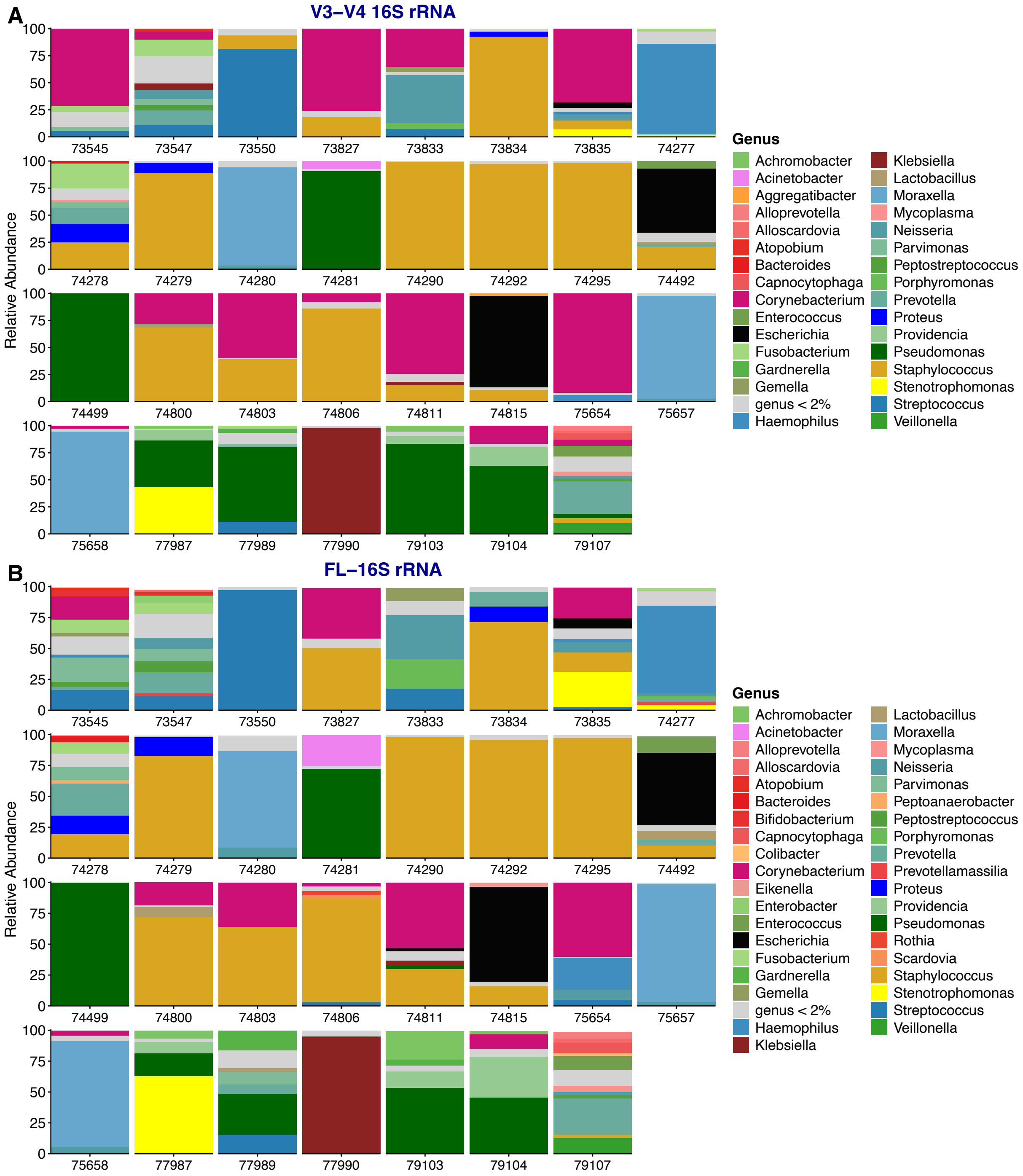

Figure S3: Genus-level taxonomic classification of tracheal aspirates. (A) V3-V4 and (B) FL-16S rRNA sequencing of 31 tracheal aspirates produce similar taxonomic profiles. Results are based on rarefied sequencing depth of 10000 reads for each type of amplicon sequencing.

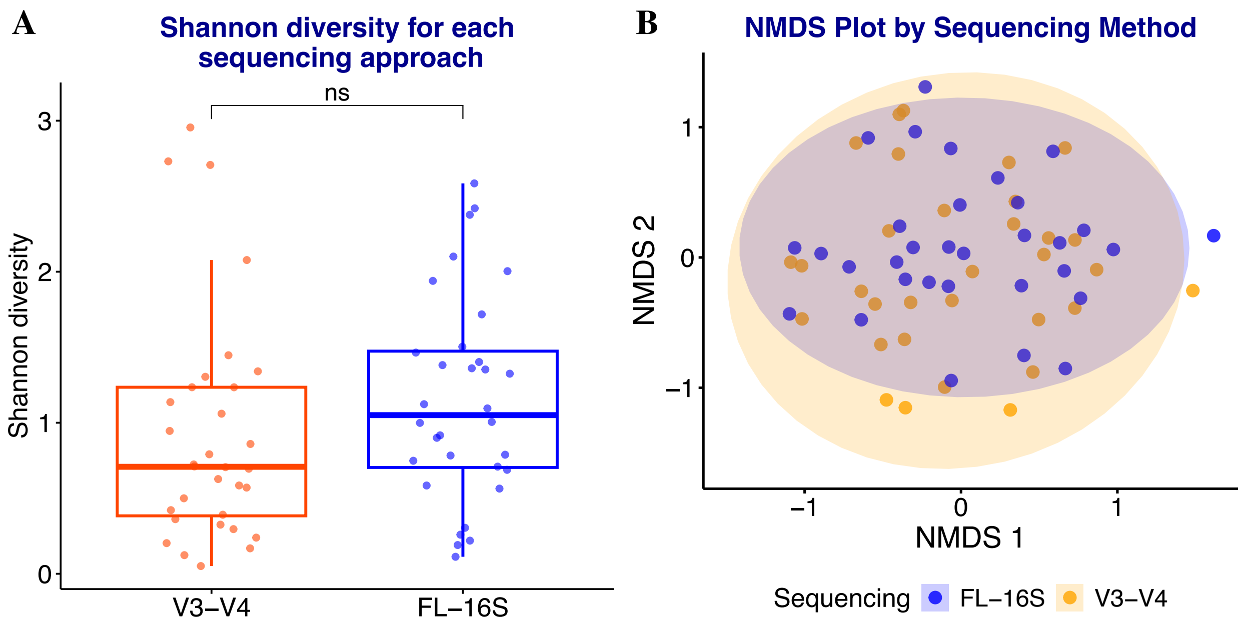

Figure S4: Community alpha and beta diversity metrics without rarefaction of reads. (A) Shannon diversity metrics comparing sequencing data of 31 tracheal aspirates using both short-read V3-V4 and long-read FL-16S rRNA sequencing. (B) FL-16S rRNA sequencing data is compositionally similar to V3-V4 sequencing data as measured using the Bray-Curtis dissimilarity index and visualized using the Non-metric multidimensional scaling (NMDS) plot. PERMANOVA, p-value = 0.76

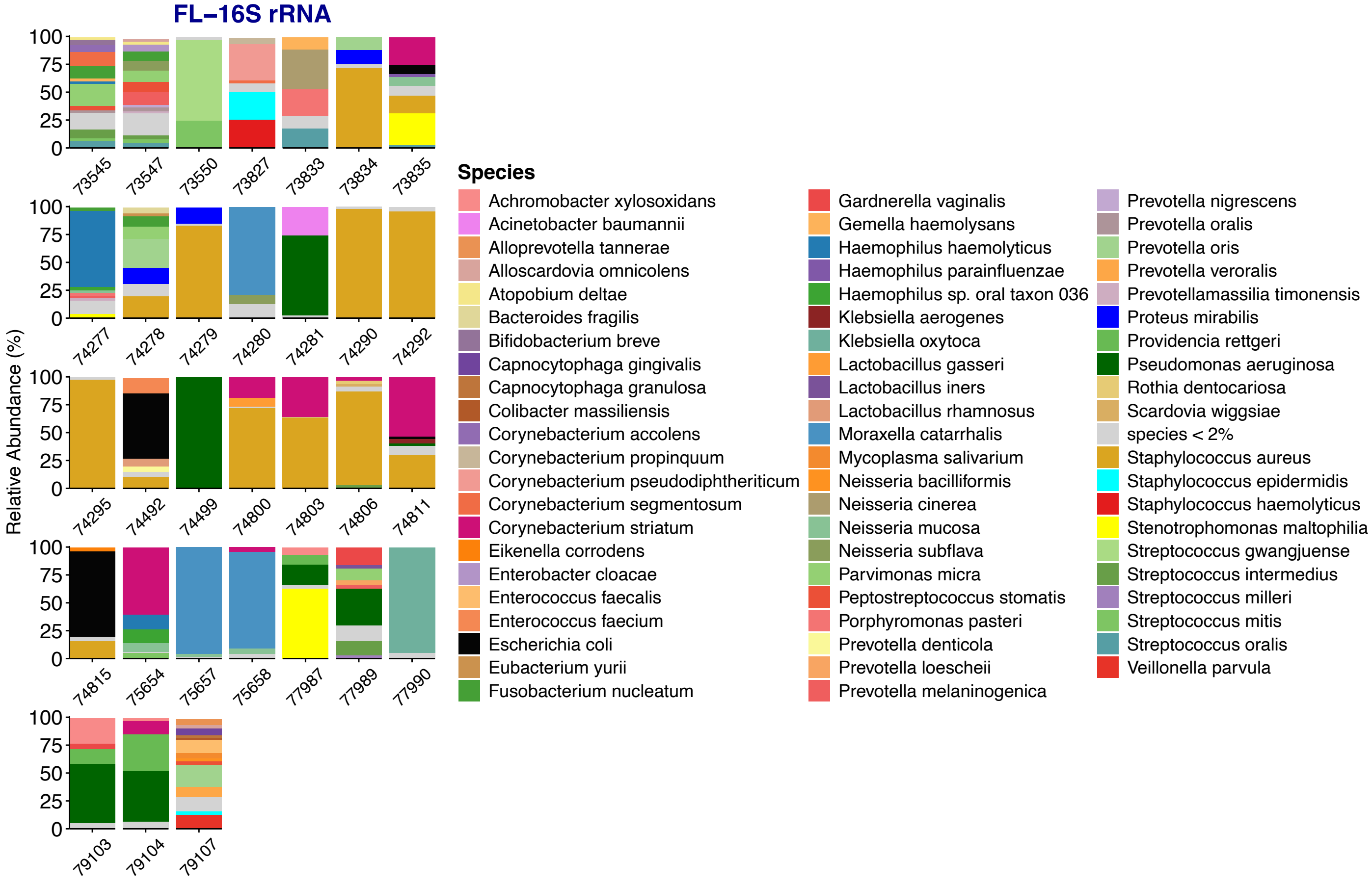

Figure S5: All species detected in tracheal aspirates using FL-16S rRNA sequencing and analyzed using Emu, using a relative abundance filter of 0.1%.
